# Multiplatform Biomarker Discovery for Bladder Cancer Recurrence Diagnosis

**DOI:** 10.1155/2016/4591910

**Published:** 2016-08-31

**Authors:** Marine De Paoli, Selma Gogalic, Ursula Sauer, Claudia Preininger, Hardev Pandha, Guy Simpson, Andras Horvath, Christophe Marquette

**Affiliations:** ^1^AXO Science SAS, 34 rue du Mail, 69004 Lyon, France; ^2^AIT Austrian Institute of Technology GmbH, Konrad-Lorenz-Straße 24, 3430 Tulln, Austria; ^3^Department of Oncology, Faculty of Health and Medical Sciences, University of Surrey, Leggett Building, Surrey GU2 7WG, UK; ^4^Université de Lyon, Université Lyon 1, CNRS, INSA, CPE-Lyon, ICBMS, UMR 5246, No. 43, boulevard du 11 Novembre 1918, 69622 Villeurbanne Cedex, France

## Abstract

*Purpose.* Nonmuscle invasive bladder cancer (BCa) has a high recurrence rate requiring lifelong surveillance. Urinary biomarkers are promising as simple alternatives to cystoscopy for the diagnosis of recurrent bladder cancer. However, no single marker can achieve the required accuracy. The purpose of this study was to select a multiparameter panel, comprising urinary biomarkers and clinical parameters, for BCa recurrence diagnosis.* Experimental Design.* Candidate biomarkers were measured in urine samples of BCa patients with recurrence and BCa patients without recurrence. A multiplatform strategy was used for marker quantification comprising a multiplexed microarray and an automated platform for ELISA analysis. A multivariate statistical analysis combined the results from both platforms with the collected clinical data.* Results.* The best performing combination of biomarkers and clinical parameters achieved an AUC value of 0.91, showing better performance than individual parameters. This panel comprises six biomarkers (cadherin-1, IL-8, ErbB2, IL-6, EN2, and VEGF-A) and three clinical parameters (number of past recurrences, number of BCG therapies, and stage at time of diagnosis).* Conclusions.* The multiparameter panel could be a useful noninvasive tool for BCa surveillance and potentially impact the clinical management of this disease. Validation of results in an independent cohort is warranted.

## 1. Introduction

Bladder cancer is the ninth most common cancer worldwide and the seventh most common cancer in men with a worldwide age-standardized rate of 9.0 per 100 000 men [1]. The majority of newly diagnosed cases are nonmuscle invasive disease (BCa) with 75% to 85% of patients presenting tumors confined to the mucosa or submucosa (Ta, carcinoma* in situ* (CIS), or T1 tumors) [2, 3]. Despite the good prognosis of such tumors, there is a tendency for recurrence after initial treatment. The probability of recurrence within 5 years ranges from 30% to 80% and 10% to 30% of these cases will progress to muscle invasive disease [3–5].

Follow-up is thus an essential aspect of BCa patient management and includes ongoing monitoring for recurrence detection. Cystoscopy and urinary cytology are defined as the gold standard methods for both diagnosis and surveillance of BCa [2]. Cystoscopy is highly sensitive but is still associated with a significant false negative rate. Moreover, as a costly, invasive, and uncomfortable procedure, it contributes to the economic and psychological burden of BCa [6, 7]. Urinary cytology has a higher specificity ranging from 85% to 100% and a high sensitivity in high-grade tumors but it lacks sensitivity in low-grade tumors [2, 8].

The management of patients with primary BCa diagnosis and postsurgical surveillance could greatly benefit from new, noninvasive methods with improved sensitivity and specificity. Urinary products of cancer growth or metabolism are highly relevant, easy to obtain, and suitable for BCa screening in these contexts. Urinary tests for diagnosis and detecting recurrence have already been developed, including FDA-approved BTA assays (BTA TRAK® and BTA stat® from Polymedco) as well as the Alere NMP22® BladderChek® Test which are used for the diagnosis and monitoring of BCa in conjunction with standard diagnostic procedures. They yield improved sensitivity (up to 89%) compared to urinary cytology which has a median sensitivity of only 35%. However, benign urological conditions tend to influence the specificity of these tests. They show a lower specificity than urinary cytology: the median specificity of BTA TRAK, BTA stat, and NMP22 is, respectively, 66%, 73%, and 73% whereas urinary cytology has a median specificity of 94% [8–10]. A recent review by van Rhijn et al. assessing the performance of 18 markers showed that urinary markers generally have a higher sensitivity but a lower specificity than urinary cytology [8]. In the context of BCa surveillance, the review also evaluated marker performance with regard to the detection of recurrent bladder cancer and found a lower sensitivity for most markers compared to their performance for primary disease detection. Thus, single markers are not currently suitable for incorporation into any clinical surveillance protocol to allow patients to undergo less frequent cystoscopic evaluations.

The ideal urinary test should show good performance in both sensitivity and specificity. As this is clearly not possible with single markers, combining several markers in a multiplexed assay might provide a solution for optimizing a BCa recurrence detection test.

A first (pilot) study was conducted by our group to identify a biomarker candidate set with potential clinical utility in BCa. The selection was made on the basis of a molecular disease model for BCa. The candidate markers were then evaluated in urine samples for their measurability and detectability in urine as well as their selectivity for BCa. This pilot study led to the definition of a five-biomarker panel (IL-8, MMP-9, VEGF-A, PTGS2, and EN2) which showed a better overall performance compared to individual markers [11]. As this study compared a group of BCa patients to a group of healthy donors, it only investigated the diagnostic aspect of* de novo* BCa.

In this report, we describe a second (discovery) study which aims at selecting a marker panel for BCa recurrence diagnosis. Biomarkers selected in the pilot study as well as other relevant candidate markers of the molecular disease model were evaluated in urine samples of BCa patients with recurrence and BCa patients without recurrence. Marker measurement was performed on two different platforms: a multiplexed microarray (BCa chip) and an automated platform for 96-well plate ELISA analysis. Results from both platforms were combined with clinical parameters for a multivariate statistical analysis allowing the selection of a panel of biomarkers and clinical parameters for the diagnosis of BCa recurrence.

## 2. Materials and Methods

### 2.1. Study Design

**Figure 1:**
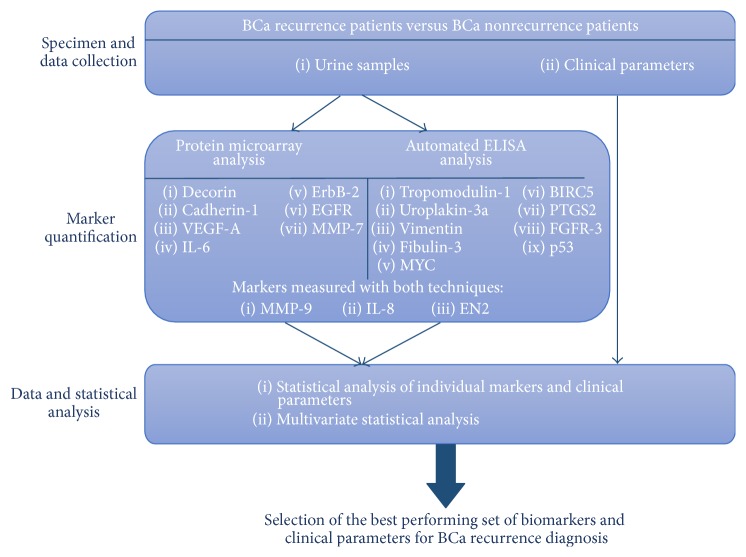
Discovery study design.

The study design is described in Figure 1. We had access to a biobank holding 80 samples from BCa patients. In total, 19 markers were measured. Two analysis platforms were used for the marker measurements and the results were included in a statistical analysis with the patients' clinical parameters. Each study step is further described in the sections below.

### 2.2. Specimen and Data Collection

The selection of patients was based on the following inclusion criteria: the patient was diagnosed with BCa (cystoscopic and histological evidence of BCa), the patient was treated for BCa prior to the sample collection visit, and the patient has no history of muscle invasive BCa. Recurrence-negative patients are defined as showing no cystoscopic or histological evidence of BCa during monitoring after treatment of* de novo* BCa. Recurrence-positive patients are defined as showing cystoscopic and histological evidence of BCa during monitoring after treatment of* de novo* BCa.

First pass urines were collected from the selected bladder cancer patients according to a standard operating procedure and were spun at 150 g for 10 minutes. The supernatant was aliquoted into 1 mL samples and frozen and stored at −80°C. All individuals gave informed consent for sample donation and the collection was approved by local ethical committee (ref. 3/LO/0739).

The samples were collected during a monitoring visit. For all samples, at least two monitoring visits are available: one for the first diagnosis and one for the point in time the sample was collected. Up to six additional visits between first diagnosis and time of sample and up to four visits after sample collection have been recorded. During the monitoring visits, the following parameters were collected: gender, age, smoking status, date, grade, stage, recurrence, TURBT (transurethral resection of the bladder tumor), and drug treatment (name and start date).

Further clinical data was collected for the statistical analysis and parameter performance regarding BCa recurrence diagnosis. Thus, the clinical parameter set used includes the following parameters: gender, age at time of diagnosis (*age.diagnosis*), age at time of sample collection (*age.sample*), time between the first diagnosis and sample collection (*months.diagnosis2sample*), number of past recurrences between the first diagnosis and time of sample collection (*no.past.recurrences*), number of bacillus Calmette-Guerin (BCG) therapies between the first diagnosis and time of sample collection (*BCG.therapy*), number of mitomycin therapies between the first diagnosis and time of sample collection (*mitomycin.therapy*), and number of transurethral resections of the bladder tumor between the first diagnosis and time of sample collection (*no.past.TURBTs*).

According to availability of the full set of clinical phenotype parameters and availability of valid biomarker readouts, 45 samples from the initial biobank of 80 samples could be included in the analysis, reflecting 27 patients being recurrence negative and 18 patients being recurrence positive.

### 2.3. Marker Quantification

Marker quantification was performed on two separate platforms. The first platform, a protein microarray (BCa chip), was used to measure the urinary levels of 10 markers, decorin, VEGF-A, cadherin-1, IL-6, ErbB2, EGFR, MMP-7, MMP-9, IL-8, and EN2, while the second platform, an automated platform for 96-well plate ELISA analysis, was used for the measurements of the following 12 markers: PTGS2, FGFR-3, uroplakin-3a, vimentin, MYC, tropomodulin-1, BIRC5, fibulin-3, p53, MMP-9, IL-8, and EN2. Three markers (MMP-9, IL-8, and EN2) were thus measured with both methods in order to evaluate the concordance of the two analytical platforms.

#### 2.3.1. BCa Chip

*(1) Materials and Reagents.* ARChip Epoxy (EP 02799374; US 10/490543) was used as assay platform. Recombinant human VEGF-A (293-VE-050), human VEGF-A antibody (MAB293) and human VEGF-A biotinylated antibody (BAF293), recombinant human MMP-7 (907-MP-010), human MMP-7 antibody (clone 111439, MAB9072), human MMP-7 biotinylated antibody (BAF907), recombinant human MMP-9 (CF, 911-MP-010), human MMP-9 antibody (MAB936), human MMP-9 biotinylated antibody (BAF911), recombinant human EGFR (1095-ER-002), human EGFR antibody (AF231), human EGFR biotinylated antibody (BAF231), recombinant decorin (143-DE-100), human decorin antibody (BAM1432) and human biotinylated decorin antibody (clone 115413, BAM1431), recombinant human ErbB2 (FC chimera, CF, 1129-ER-050), human ErbB2 antibody (clone 191924, MAB1129), human ErbB2 biotinylated antibody (BAF1129), recombinant human cadherin-1 (648-EC-100), human cadherin-1 antibody (clone 77308, MAB18382), and human cadherin-1 biotinylated antibody (BAF648) were derived from R&D Systems (Minneapolis, MN). Recombinant human IL-6 (14-8069-80), human IL-6 antibody (14-7069-85), and human IL-6 biotinylated antibody (13-7068-85) were purchased from eBioscience (San Diego, CA). The aptamer for EN2 (5′-/5ThioMC6-D/AA AAA AAA AAC GCA TAA TTA CCT CCA GAA GGA GAG GTA ATT ATG CG-3′, HPLC purified) [12] was obtained from IDT Integrated DNA Technologies (Coralville, IA). Human EN2 (orb16876) was derived from Biorbyt (Cambridge, UK). Human EN2 biotinylated antibody (bs-11552R-biotin) was from BioSS (Woburn, MA). Recombinant human IL-8 (574202), human IL-8 antibody (clone H8A5, 511501), and human IL-8 biotinylated antibody (clone E8N1, 511404) were purchased from Biolegend (San Diego, CA). CRP/Dy647 (CON5, clone 7, D603080) was purchased from Exbio (Vestec, Czech Republic). LowCross-Buffer (100 050) from Candor (Wangen, Germany) was used as assay buffer. Dy647 streptavidin was obtained from Dyomics (Jena, Germany) and Tween 20 and sodium deoxycholate were derived from Sigma-Aldrich (Vienna, Austria). Certified drug-free urine (88121-CDF(F)) was used from UTAK Laboratories (Valencia, CA).

*(2) Chip Fabrication and Processing.* Capture antibodies were diluted in spotting buffer (1 × PBS (pH 7.2)/0.01% sodium deoxycholate) to concentrations of 0.4 mg/mL for MMP-9, decorin, VEGF-A, IL-8, IL-6, MMP-7, cadherin-1, ErbB2, and 0.16 mg/mL for EGFR. The concentration of spotted aptamer EN2 was 100 *μ*M. The capture elements were printed on proprietary ARChip Epoxy glass slides using the Arrayit Nanoprint contact spotter from GeneMachines (pin SMP3). The spot-to-spot distance was 350 *μ*m. Each probe was spotted in triplicate in 12 identical arrays at a relative humidity of 50% and kept at 4°C for a minimum of three days. The arrayed slides were then blocked for 30 min in 1 × PBS (pH 7.2)/0.1% Tween 20 and washed twice in 1 × PBS (pH 7.2). For one experiment, a 4-slide set was mounted into an Arrayit Hybridization Cassette 4 × 16. The frames create an incubation well for each array (7 × 7 mm). We used 12 arrays per slide, resulting in 48 arrays per slide set. The sets accommodate 27 replicate measurements (three arrays with three replicate spots and three repeats) of 10 calibration standards and 45 urine samples. Standard curves for each marker were generated in synthetic urine diluted with assay buffer (1 : 3). Each array was incubated with 50 *μ*L calibration standard or sample for 2.5 hours. After washing three times with 1 × PBS (pH 7.2)/0.1% Tween 20, slides were incubated for 45 min with 50 *μ*L biotinylated antibody mixes (1 *μ*g/mL). Then, the slides were again washed and incubated with 4 *μ*g/mL Dy647 streptavidin. At the end, the slides were washed twice with 1 × PBS (pH 7.2)/0.1% Tween 20 and twice in 1 × PBS (pH 7.2).

All incubation steps were carried out on the orbital shaker at room temperature. The slides were stored in the dark until scanning.

*(3) Data Analysis.* Processed slides were scanned using a GenePix 4000B nonconfocal scanner (Molecular Devices, Sunnyvale, CA) (*λ*_ex_: 635 nm, *λ*_em_: 670 nm) and data were analyzed with the GenePix 6.0 software. The photomultiplier tube (PMT) voltage was held constant throughout the scans for each analyte to allow data comparison. The mean signal values were calculated from 27 background corrected data points. Data that were out of the mean signal values ± the standard deviation (SD) were excluded. Standard curves were set up with GraphPad Prism 5 with a logistic fit.

#### 2.3.2. Automated Platform for 96-Well Plate ELISA Analysis

Regarding the second platform, commercially available ELISA kits were used to measure the markers' urinary levels. Supplemental Table  1 (see Supplementary Table  1 in Supplementary Material available online at http://dx.doi.org/10.1155/2016/4591910) gives the kits sources and references. The automated analysis was performed using an EVO100 robotic platform (Tecan, Männedorf, Switzerland). Each assay was conducted according to the manufacturer's instructions and included a calibration curve in assay diluent as well as a calibration curve in normal human urine (UTAK Laboratories, Valencia, CA). Both were prepared using protein standards provided in the ELISA kits. Four-parameter logistic regression was used for curve fitting.

### 2.4. Data and Statistical Analysis

#### 2.4.1. Sample Selection

Before the statistical analysis, further sample selection was performed in order to use clinical data most stringent with respect to recurrence events in BCa. A complete history (no missing clinical parameters) is thus required. Based on this additional criterion, 48 of the initial 80 patients were included. Furthermore, three other samples had to be excluded during the biomarker selection process due to lack of valid biomarker readout (see section below) leading to a final study cohort of 45 patients (27 nonrecurrence BCa patients and 18 recurrence BCa patients).

#### 2.4.2. Biomarker Selection and Preprocessing

In total, 19 markers were measured in the study. To avoid artifacts caused by missing value imputation, biomarkers were required to have technically valid measurements for all samples in the analysis. Biomarker candidates for the current analysis were selected in such a manner that both the number of included biomarker candidates and the number of included samples are maximized. Under these constraints, a set of 13 biomarker candidates with complete measurements for 45 out of the 48 samples was identified. Ten of the 13 markers were measured with the first platform: decorin, VEGF-A, IL-8, cadherin-1, IL-6, EN2, EGFR, ErbB2, MMP-7, and MMP-9. Three were measured with the second platform: IL-8, MMP-9, and fibulin-3.

Measurements below or above the valid range were set to respective lower and upper detection limit values.

#### 2.4.3. Individual Parameter Performance

Descriptive statistics for the two patient groups for the individual clinical parameters and individual biomarker candidates were performed. In addition, their discriminative performance for the two patient groups were evaluated using* Z* statistic.

#### 2.4.4. Multivariate Statistical Analysis

As the aim of this study was to determine a classifier for recurrence risk, multimarker panels (composed of clinical parameters as well as molecular readouts) were included in multivariable regression models. Two strategies were employed for constructing these models: (i) manual selection based on individual parameter association with the outcome parameter and given clinical evidence and (ii) automatic selection using Least Absolute Shrinkage and Selection Operator (LASSO) regression for determining selection probability of individual parameters.

For the selected parameter sets, a generalized linear model (GLM) for predicting BCa recurrence was constructed on the basis of given sample parameters. Receiver operator characteristics (ROC) along with the correlation matrix for model parameters were determined. Model performance in terms of AUC was also determined. In addition to training AUC values, leave-one-out cross-validation (LOOCV) AUC values were determined.

## 3. Results

### 3.1. Biomarkers Measurements

Ten markers were analyzed in 1 : 3 diluted patient's urine using the BCa chip. The assay sensitivity for each marker was visually determined and is displayed in Table 1.

Highest assay sensitivity was observed for decorin (LOD 45 pg/mL). Best reproducibility was achieved for markers IL-6 and EN2 (CV 7%). Limits of detection (LOD), linear ranges, and CV of some BCa markers were also described in previous articles ([13] and submitted paper: [14]).

Twelve markers were measured in patient samples using the automated platform for 96-well plate ELISA analysis. The results are summarized in Table 2. No measurement was obtained for uroplakin-3a as the full analysis could not be carried out due to a technical difficulty. All other assays except for fibulin-3 provided a calibration curve in standardized urine. Fibulin-3 concentrations in samples were thus determined using the calibration curve in assay diluent. Three out of the 11 valid assays were not able to detect their specific target in patient urine samples (vimentin, MYC, and p53). Our previous study has shown similar results for vimentin and p53. Moreover, although MYC was detected in patient samples, the study's ROC analysis ranked MYC as the lowest performing marker [11].

As it was mentioned in* Biomarker Selection and Preprocessing*, further statistical analysis was only performed on three selected biomarkers: IL-8, MMP-9, and fibulin-3.

Two markers, namely, IL-8 and MMP-9, were measured by both analytical systems. Significant positive correlations were observed for both valid markers with Pearson *R* values of 0.83 for IL-8 and 0.70 for MMP-9.

### 3.2. Individual Parameters Performance

#### 3.2.1. Clinical Parameters

In order to evaluate the potential of individual clinical parameters for diagnosing BCa recurrence, these parameters were compared between the two patient groups. Each parameter was also evaluated in terms of discriminative performance for BCa recurrence (Table 3).

No apparent variance is detected in clinical parameters allowing for the prediction of BCa recurrence. However, even though not significant individually, the parameters* no.past.recurrences*,* BCG.therapy*, and* no.past.TURBTs* appear to exhibit some association with the target parameter. These results clearly indicate the need of including additional molecular biomarkers for improving diagnostic accuracy.

#### 3.2.2. Biomarker Candidates

For each individual biomarker, measurements were compared between the nonrecurrent group and the recurrent group and their discriminative performance is provided in Table 3.

From this analysis, no single biomarker candidate stands out with the desired performance for a recurrence diagnostic test. The highest AUC value, 0.7305, was found for ErbB2. Some markers show AUC values lower than 0.5 (IL-6_chip_, MMP-7_chip_, IL-8_AP_, and MMP-9_AP_). The other markers show AUC values ranging from 0.5247 to 0.6872. This lack of performance clearly emphasizes the need to use a profile of parameters in order to achieve acceptable diagnostic performance. The most promising markers in terms of individual performance (VEGF-A_chip_, IL-8_chip_, EN2_chip_, and ErbB2_chip_) were thus forwarded to multivariate analysis.

### 3.3. Combined Parameters Performance

As the individual performances of the clinical parameters and the biomarker candidates did not reach a sufficient level, various combinations of both types of parameters were evaluated to define a multiparameter panel. Six multivariate regression models were constructed (see Table 4). The corresponding correlation matrix and the receiver operator characteristics as well as the models' AUC values are provided in Table 4.

Constructed models based on automatically selected parameters show overall superior performance over the models consisting of manually selected parameters. In case of the clinical parameter models (Model 1 and Model 2), the automatically determined model (Model 2) holds one parameter less than the manually selected model but still outperforms the manually constructed model. The drop in the LOOCV AUC of Model 1 may be partially attributed to highly correlated predictor variables* no.past.recurrences *and* no.past.TURBTs*. Given the fact that Model 4 holds more parameters than Model 3, the higher training AUC is not surprising. However, Model 4 also exceeds Model 3 in terms of the LOOCV AUC and appears therefore to be more suited for diagnosing BCa recurrence. Similarly, Model 6 comprising parameters from the automatically constructed clinical parameters and biomarker candidates models supersedes its counterpart Model 5. Again, as Model 6 holds one parameter more, the increase in the training AUC (Model 5: 0.82; Model 6: 0.91) was to be expected. Nevertheless, we also observed an increase in the LOOCV AUC (Model 5: 0.64; Model 6: 0.7). Considering all the models presented here, Model 6 shows the best performance in terms of AUC and reaches an AUC of 0.70 in cross-validation.

## 4. Discussion

Due to the high rate of BCa recurrence, surveillance is a key element of patient management. Thus, great clinical benefit could be gained from improved tools for evidence-based risk assessment, which would influence surveillance strategy. The European Organization for Research and Treatment of Cancer (EORTC) has developed a scoring system to predict short- and long-term risks of disease recurrence and progression after transurethral resection. This scoring system is solely based on conventional clinical and pathological predictors of outcomes (tumor stage and grade, number of tumors, tumor size, concomitant CIS, and history of prior disease recurrence) [15]. EORTC risk tables show significant limitations and its risk classification could be improved by the updating and addition of new parameters [16–18]. According to a comprehensive review of the literature by Kluth et al. regarding the prognostic and prediction tools in BCa, clinical utility and accuracy of such tools could be improved by biomarkers [17].

Only a few studies have taken into consideration biomarkers as parameters of prediction tools. One specific study identified a panel of 5 cell cycle regulatory biomarkers (cyclin E1, p53, p21, pRB, and p27) which improved the predictive accuracy of BCa recurrence and survival after cystectomy in patients with pTa-3N0M0 tumors [19]. In another study, Rosser et al. evaluated the performance of a 10-biomarker panel (IL-8, MMP-9, MMP-10, SERPINA1, VEGF-A, ANG, CA9, APOE, SERPINE1, and SDC1) which achieved better sensitivity (79%) than urine cytology (33%) for recurrent BCa detection and outperformed any single biomarker [20].

In our discovery study, we evaluated the potential of several biomarkers and clinical parameters for BCa recurrence diagnosis. Individual parameter performance analysis showed that clinical data alone is not sufficient for the diagnosis of recurrence. Moreover, no single biomarker candidate achieved an acceptable performance by itself. By combining both types of parameters, clinical and molecular, we identified several multivariable regression models. The best performing model (Model 6) achieved an AUC of 0.91, showing better performance than the single parameters. It comprises the following automatically selected (using LASSO) clinical parameters and biomarker candidates: number of past recurrences, number of BCG therapies, stage at time of diagnosis, cadherin-1, IL-8, ErbB2, IL-6, EN2, and VEGF-A.

Regarding clinical parameters, all of the manually selected parameters are also included in Model 6 apart from age at time of sample and number of TURBTs. The parameter age at time of sample was included during manual selection based on its presumed clinical relevance and was not based on observed statistical association with the target parameter. Number of TURBTs is highly correlated with number of past recurrences and should therefore be omitted.

All the biomarkers identified as relevant in the individual analysis are included in the selected model (IL-8, ErbB2, EN2, and VEGF-A). The multivariate analysis provided two additional markers: IL-6 and cadherin-1. All six biomarkers were previously proved to be of interest with regard to BCa recurrence. Indeed, a study suggested that assessment of cytoplasmic cadherin-1 staining can predict time to BCa recurrence [21] while Mahnken et al. found a correlation between abnormal cadherin-1 immunostaining and early tumor recurrence, identifying this parameter as an independent predictor of recurrence-free survival [22]. A relationship between high expression levels of IL-8 in malignant tissues and tumor recurrence was also found [23]. A similar finding was described for IL-6 [24]. Moreover, a cytokine panel including both IL-8 and IL-6 showed potential in tumor recurrence risk identification in patients undergoing BCG treatment [25]. Several studies revealed the correlation between VEGF-A and BCa recurrence, at the mRNA level as well as the protein level [26–28]. As for ErbB2, its overexpression has been associated with BCa and risks of recurrence and progression but it is not yet clearly established as a molecular marker in recurrent bladder tumors as some studies showed varying results [29–32]. Finally, EN2 has been studied as a potential urinary marker for BCa diagnosis but was also shown to be secreted by recurrent nonmuscle invasive BCa tumors [33].

Although links to recurrence or its mechanism are not clearly established yet for all the selected biomarkers, they all show a potential role for revealing BCa recurrence. Combining these markers with clinical parameters appears to be a good strategy for achieving acceptable performance with regard to BCa recurrence diagnosis. Such a panel will allow the determination of patient-specific profiles and could greatly improve recurrence detection.

Although our findings show potential for the development of new BCa surveillance tool, some limitations in our study need to be considered. Indeed, the study includes only a small number of samples due to stringent selection criteria. Furthermore, the study design only allowed evaluating the diagnosis of BCa recurrence, giving no information on the prediction of BCa recurrence. Validation of our results in an independent cohort shall be warranted.

## 5. Conclusions

In conclusion, cancer is a complex disease and as its detection cannot rely on a single biomarker, the analysis of a profile of biomarkers will likely be more accurate for such purpose [34]. The challenge remains thus in selecting those biomarkers which reflect early tumor growth and disease activity. Our multiplatform strategy allowed the screening of 19 urinary markers for BCa recurrence diagnosis and led to a selection of six markers as well as three clinical parameters defining a panel for patient segmentation. This multiparameter panel outperformed any single biomarker or clinical parameter for BCa recurrence diagnosis and could be a useful tool for BCa surveillance scheme.

However, our discovery study shows limitations especially in terms of number of patients. The total number of patient samples included in the statistical analysis was reduced by our selection sample method. Moreover, our study only evaluated the diagnostic aspect of recurrence in nonmuscle invasive BCa patients and did not take into consideration the prediction aspect. According to the IBCN phases classification for the development of diagnostic markers in bladder cancer, our study corresponds to a phase II study, evaluating clinical utility [35]. Further clinical validation of the panel is thus needed, in a phase III study. A larger independent validation study would allow the confirmation of our findings and lead to the design of a novel patient stratification concept as a risk estimating tool. Clinical management of BCa, especially surveillance, could greatly benefit from stratification incorporating patient-specific biomarker and clinical profiles.

## Supplementary Material

Supplementary material describes the content of each marker assay kit together with their source and characteristics.

## Figures and Tables

**Table 1:** Biomarker measurements using the BCa chip.

Marker	LOD (pg/mL)	CV (%)
Decorin	45	11
VEGF-A	810	23
IL-8	405	9
Cadherin-1	2430	11
IL-6	135	7
EN2	729	7
ErbB2	450	18
EGFR	135	15
MMP-7	810	12
MMP-9	1350	14

LOD: limit of detection.

**Table 2:** Biomarker measurements with the automated platform.

Marker	Calibration curve in assay diluent	Calibration curve in urine	LOD (urine)	Marker detection in samples
PTGS2	−	+	5 ng/mL	+
FGFR-3	+	+	1000 pg/mL	+
Uroplakin-3a	−	−	N/A	N/A
Vimentin	+	+	112.5 ng/mL	−
MYC	+	+	1.25 ng/mL	−
Tropomodulin-1	+	+	2.5 ng/mL	+
BIRC5	+	+	1000 pg/mL	+
Fibulin-3	+	−	25 ng/mL (assay diluent)	+
p53	+	+	10 ng/mL	−
MMP-9	+	+	666.7 pg/mL	+
IL-8	+	+	125 pg/mL	+
EN2	+	+	1.25 ng/mL	+

+: presence of calibration curve or marker detection in samples.

−: absence of calibration curve or no marker detection in samples.

N/A: not applicable; LOD: limit of detection.

**Table 3:** Discriminative performance of individual clinical parameters and biomarker candidates for BCa recurrence.

Clinical parameter	Pr (>|*z*|)	AUC	Biomarker candidate	Pr (>|*z*|)	AUC
diagnosis2sample	0.42	0.50	Decorin_chip_	0.67	0.57
gender	0.89	0.51	VEGF-A_chip_^*∗*^	0.05	0.67
age.diagnosis	0.70	0.48	IL-8_chip_^*∗*^	0.08	0.69
age.sample	0.45	0.57	Cadherin-1_chip_	0.66	0.53
grade.diagnosis (G2/G3)	0.32/0.48	0.57	IL-6_chip_	0.65	0.48
stage.diagnosis	0.52	0.55	EN2_chip_^*∗*^	0.09	0.65
no.past.recurrences^*∗*^	0.08	0.63	EGFR_chip_	0.86	0.53
BCG.therapy^*∗*^	0.10	0.65	ErbB2_chip_^*∗*^	0.06	0.73
mitomycin.therapy	0.49	0.54	MMP-7_chip_	0.90	0.50
no.past.TURBTs	0.33	0.58	MMP-9_chip_	0.72	0.58
			IL-8_AP_	0.74	0.47
			MMP-9_AP_	0.46	0.50
			Fibulin-3_AP_	0.54	0.52

^*∗*^Clinical parameters and biomarker candidates with the best individual AUC.

(a)* grade.diagnosis*: tumor grade at time of diagnosis; *stage.diagnosis*: tumor stage at time of diagnosis. The other clinical parameters are defined in the *Specimen and Data Collection*.

(b) G2/G3: grade 2/grade 3.

(c) Biomarkers ending with *chip *were measured with the BCa chip and markers ending with *AP *were measured with the automated platform for 96-well plate ELISA analysis.

**Table 4:** Multivariate regression models.

Model	Strategy	Description	Included parameters	AUC	AUC (LOOCV)
Model 1	Manual selection	The model comprises clinical parameters exhibiting on the individual level some association with the outcome parameter and the clinically relevant age at time of sample	no.past.recurrences, BCG.therapy, no.past. TURBTs, and age.sample	0.78	0.65
Model 2	Automatic selection	The model comprises clinical parameters with a selection probability greater than 50%	no.past.recurrences, BCG.therapy, and stage.diagnosis	0.80	0.72
Model 3	Manual selection	The model comprises biomarker candidates exhibiting on the individual level some association with the outcome parameter	VEGF-A_chip_, IL-8_chip_, EN2_chip_, and ErbB2_chip_	0.72	0.51
Model 4	Automatic selection	The model comprises biomarker candidates with a selection probability greater than 50%	Cadherin-1_chip_, IL-8_chip_, ErbB2_chip_, IL-6_chip_, EN2_chip_, and VEGF-A_chip_	0.78	0.61
Model 5	Union of the parameters in Model 1 and Model 3			0.82	0.64
Model 6	Union of the parameters in Model 2 and Model 4			0.91	0.70

(a) Included parameters:

*stage.diagnosis*: stage of the tumor at time of diagnosis.

The other clinical parameters are defined in the *Specimen and Data Collection*.

(b) Markers ending with *chip *were measured with the BCa chip and markers ending with *AP *were measured with the automated platform for 96-well plate ELISA analysis.

(c) LOOCV: leave-one-out cross-validation.

(d) Biomarker candidates chosen during manual selection for Model 3 are a subset of Model 4.
