# Supplementary material for: Multiplatform Biomarker Discovery for Bladder Cancer Recurrence Diagnosis
Source: Dis Markers. 2016 Aug 31;2016:4591910. doi: 10.1155/2016/4591910 (PMC5021863; doi:10.1155/2016/4591910)
Supplement: Supplementary file 1 — Supplementary material describes the content of each marker assay kit together with their source and characteristics. [file 4591910.f1.docx]

Supplemental Table  1. ELISA kits references.

| Marker full name (symbol) | Company | Cat No |
| --- | --- | --- |
| Prostaglandin-endoperoxide synthase 2 (PTGS2) | Uscn Life Science | SEA699Hu |
| Fibroblast growth factor receptor 3 (FGFR-3) | Cusabio | CSB-EL008646HU |
| Uroplakin 3A | Cusabio | CSB-EL025657HU |
| Vimentin | Abnova | KA3127 |
| V-Myc myelocytomatosis viral oncogene homolog (MYC) | BlueGene Biotech | E01C0774 |
| Tropomodulin-1 | Cusabio | CSB-EL023909HU |
| Baculoviral IAP repeat containing 5 (BIRC5) | Abnova | KA0441 |
| Fibulin-3 | USCN Life Science | SEF422Hu |
| Cellular tumor antigen p53 (p53) | Sigma-Aldrich | RAB0500-1KT |
| Matrix metalloproteinase-9 (MMP-9) | Abcam | ab100610 |
| Interleukin-8 (IL-8) | Abcam | ab46032 |
| Homeobox protein engrailed-2 (EN2) | Cusabio | CSB-EL007660HU |
